# Influence of parental weight change on the incidence of overweight and obesity in offspring

**DOI:** 10.1186/s12887-022-03399-8

**Published:** 2022-06-07

**Authors:** Hui Fan, Xingyu Zhang

**Affiliations:** 1 Department of Epidemiology and Health Statistics, School of Public Health, North Sichuan Medical College, No. 234 Fujiang Road, Shunqing District, Nanchong, 637000 China; 2 Thomas E.Starzl Transplantation Institute, University of Pittsburgh Medical Center, Pittsburgh, USA

**Keywords:** Overweight, Obesity, Offspring, Parent

## Abstract

**Background:**

There is limited information on the association of parental weight change with overweight and obesity in offspring. This study aimed to investigate the association between parental weight change and incident overweight and obesity in offspring.

**Methods:**

This longitudinal cohort study included 2,963 parent–offspring trios who participated in at least two waves of the China Health and Nutrition Survey. The children without overweight and obesity defined by the International Obesity Task Force were included at the initial survey. Parental overweight and obesity were defined as body mass index ≥ 25 kg/m^2^.

**Results:**

The incidence of overweight and obesity in offspring was 5.8% during a mean follow-up of 5.4 years. Paternal and maternal overweight and obesity at baseline were associated with this condition in offspring at follow-up (both *P*_s_ < 0.05). Compared with the persistent normal group, the persistent overweight and obesity group and incident overweight and obesity group (normal weight to overweight and obesity), but not the reversion group (overweight and obesity to normal weight), were more likely to report overweight and obesity in offspring at follow-up, regardless of father’s or mother’s condition. Additionally, compared with offspring whose both parents remained normal weight, those whose both parents changed from overweight and obesity to normal weight or whose one parent changed from overweight and obesity to normal weight while the other remained normal weight had no higher risks of overweight and obesity.

**Conclusion:**

This study highlights the importance of parental weight management in the prevention of overweight/obesity in offspring.

**Supplementary Information:**

The online version contains supplementary material available at 10.1186/s12887-022-03399-8.

## Background

The prevalence of overweight and obesity in adults and children has rapidly increased in most countries over the past two decades [1–3]. This global overweight and obesity epidemic has been hard to control given that this condition develops in early life and persists into middle and late life [4]. Consequently, prevention of childhood overweight and obesity has become a global priority.

To develop effective strategies to prevent overweight and obesity in early life, the major and modifiable risk factors for these conditions need to be determined. Most previous studies have focused on children’s own risk factors for overweight and obesity [5]. However, the transmission of overweight and obesity across generations has recently gained increasing attention. Cross-sectional studies have indicated that parental overweight and obesity are positively correlated with these conditions in offspring [6, 7]. Moreover, cohort studies have confirmed the correlation of parental overweight and obesity during pre-pregnancy with the high body mass index (BMI) of their infant, adolescent, and young adult offspring [8–10]. Pre-conception parental obesity induces obesity in offspring via epigenetic mechanisms that influence gametogenesis and reprogram processes during embryogenesis and early development [11, 12]. A systematic review and meta-analysis showed that the influence of parental overweight and obesity on these conditions in offspring varied according to the economic level of the country they lived in [13]. However, evidence regarding the above-mentioned relationship in Chinese population is limited. The trends in prevalence of overweight and obesity in Chinese adults and children increase; thus, clarifying the association between the weights of the parents and offspring has become necessary [1, 2].

This study aimed to examine the association between parental change in BMI status and the incidence of overweight and obesity in offspring using a cohort study of Chinese population.

## Methods

### Study population

The China Health and Nutrition Survey (CHNS) is an open, continuous, household-based cohort study that is currently underway in China [14]. It started in 1989, with follow-up surveys conducted in 2- or 4-year intervals. A total of 10 survey waves were completed between 1989 and 2015. The CHNS uses a multistage, random-cluster design for sampling household members [14]. All methods were carried out in accordance with the declaration of Helsinki. Signed informed consent was obtained from all participants prior to each survey. The CHNS was approved by the institutional review boards of the University of North Carolina at Chapel Hill and the Institute of Nutrition and Health, Chinese Center for Disease Control and Prevention.

In total, 5,105 children aged 3–17 years who participated in at least two survey waves (range: 2–5 waves) were included in this study. At both the first and last surveys, the children were living with their apparently healthy parents, and neither the children nor their parents had any obvious outlying data (e.g., BMI > 60 kg/m^2^ or < 5 kg/m^2^). We excluded 1,794 children with parental weight or height missing at the initial or last survey. We also excluded 348 children with overweight and obesity at the initial survey to assess the influence of parental weight change on incident overweight and obesity in offspring in the cohort. Finally, 2,963 parent–offspring trios were included in the current study.

### Parental weight

Parental weight in light clothing and height without shoes were measured by trained technicians. BMI was calculated as weight in kilograms divided by height in meters squared (kg/m^2^). Parental overweight and obesity was defined as BMI ≥ 25 kg/m^2^ [15].

Changes in the maternal and paternal weight status from the first to last survey were defined as follows: (i) “persistent normal weight” if the status was normal weight at both time points, (ii) “incident overweight and obesity” if the status changed from normal weight to overweight and obesity, (iii) “reversion” if the status changed from overweight and obesity to normal weight, and (iv) “persistent overweight and obesity” if the status was overweight and obesity at both time points.

The participants were then categorized into six groups based on the following combinations of paternal and maternal weight change: (i) both parents remained normal weight; (ii) both parents’ weights changed from overweight and obesity to normal weight/one parent’s weight changed from overweight and obesity to normal weight and the other remained normal weight; (iii) one parent remained normal weight and the other developed overweight and obesity; (iv) one parent remained normal weight and the other remained to have overweight and obesity; (v) one parent’s weight changed from overweight and obesity to normal weight and the other remained to have overweight and obesity/one parent’s weight changed from overweight and obesity to normal weight and the other developed overweight and obesity; and (vi) both parents developed overweight and obesity/both parents remained to have overweight and obesity/one parent remained to have overweight and obesity and the other developed overweight and obesity (Table S1).

### Children’s weight

The weight and height of the children were measured by trained technicians, similar to that mentioned above for the parents. Overweight and obesity in offspring was defined as BMI greater than or equal to the corresponding sex- and age-specific cut-off values for overweight, as established by the International Obesity Task Force [16]. The above-mentioned sex- and age-specific cut-off values were obtained from the centile curves passed through cut-off values of 25 kg/m^2^ at age 18 years (adult overweight cut-off values) [16].

### Covariates

Sex, age, residence (urban/rural), and ethnicity (Han/others) were self-reported at each survey. In offspring, the energy intake was evaluated on the basis of three consecutive 24-h dietary recalls and household food inventories according to a Chinese food composition table [17]. The details of the dietary assessment are described in a previous study [17]. Household assets included color TV, refrigerator, microwave oven, electrical cooking pot, air conditioner, electric fan, and camera. Considering each asset worth 1 point, the household asset score ranged from 0 to 7. The length of follow-up was calculated by the gap between last and first survey year. We used mode imputation method for missing covariates information except for sex and age given corresponding missing data were less.

### Statistical analysis

We used a normality test (the Kolmogorov–Smirnov test) to identify whether the data were normally distributed. Mean ± standard deviation and median (interquartile range) were considered as normal and non-normal continuous variables, respectively. Categorical variables were presented as number (percentages).

A logistic regression model adjusted for sex, ethnicity, the length of follow-up, and baseline covariates (the year of study entry, age, residence, energy intake, and household asset score) was used to investigate the influence of baseline paternal overweight and obesity (exposure) on the incidence of overweight and obesity in offspring (outcome). Similarly, we used a covariate-adjusted logistic regression model to assess the association of baseline maternal overweight and obesity (exposure) with the incidence of overweight and obesity in offspring (outcome). We also used a covariate-adjusted logistic regression model to assess the association between the number of parents with baseline overweight and obesity (exposure) and the incident overweight and obesity in offspring (outcome).

A logistic regression model adjusted for the aforementioned covariates was also used to assess the association of paternal weight change (exposure) with the incidence of overweight and obesity in offspring (outcome). We also used a covariate-adjusted logistic regression model to assess the influence of maternal weight change (exposure) on the incidence of overweight and obesity in offspring (outcome). To evaluate the stability of these associations, we conducted the sensitivity analyses. First, stratified analyses were performed by the sex of offspring (male/female offspring). Second, the definition of overweight and obesity in Chinese adults (BMI ≥ 24 kg/m^2^) was used to identify paternal and maternal overweight and obesity [18]. The definition of overweight and obesity in Chinese children was applied to offspring as well [18]. Offspring aged less than 6 years (no cut-off for overweight provided by the definition of overweight and obesity in Chinese children for this age group) and offspring with overweight and obesity, as identified using the abovementioned definition at the initial survey, were excluded [18]. We repeated the analyses.

In addition, a covariate-adjusted logistic regression model was used to assess the influence of the various combinations of paternal and maternal weight change (exposure) on the incidence of overweight and obesity in offspring (outcome).

All analyses were performed using SAS 9.4 (SAS Institute Inc., Cary, NC), and two-tailed *P* values ≤ 0.05 were considered statistically significant.

## Results

The incidence of overweight and obesity in offspring was 5.8% during a mean follow-up of 5.4 years. The baseline characteristics of the parent–offspring trios are summarized in Table 1. The median ages of the offspring, fathers, and mothers at baseline were 7.0, 36.0, and 34.0 years, respectively. The median BMIs of the offspring, fathers, and mothers at baseline were 15.5, 21.6, and 21.5 kg/m^2^, respectively. The prevalence of paternal and maternal overweight and obesity at baseline was 13.7% and 14.3%, respectively.

**Table 1:** Baseline characteristics of participants in this study

Variables	
Household (*n* = 2963)	
Household asset score	1.0 (2.0)
Urban residence	803 (27.1%)
Year of study entry	
1991	1545 (52.1%)
1993	265 (8.9%)
1997	382 (12.9%)
2000	202 (6.8%)
2004	195 (6.6%)
2006	97 (3.3%)
2009	165 (5.6%)
2011	112 (3.8%)
Father (*n* = 2963)	
Age, years	36.0 (8.0)
BMI, kg/m^2^	21.6 (3.3)
Overweight and obesity	405 (13.7%)
Mother (*n* = 2963)	
Age, years	34.0 (8.0)
BMI, kg/m^2^	21.5 (3.6)
Overweight and obesity	425 (14.3%)
Children (*n* = 2963)	
Male	1536 (51.8%)
Age, years	7.0 (6.0)
Han Nationality	2524 (85.2%)
Energy intake, kcal/day	1669.6 (851.0)
BMI, kg/m^2^	15.5 (2.1)
Overweight and obesity	0

The normal and non-normal continuous variables were described as mean ± standard deviation and median (interquartile range), respectively. Categorical variables were presented as number (percentages)

BMI indicates body mass index

Table S2 presents the parental baseline weight and risk of overweight and obesity in offspring. Paternal and maternal overweight and obesity at baseline were significantly associated with these conditions in offspring at follow-up (all *P*_s_ < 0.05). Children who had one or both parents with overweight and obesity at baseline were more likely to have overweight and obesity at follow-up than those whose both parents had normal weight (all *P*_s_ < 0.05). A significant increase trend was noted in the prevalence of overweight and obesity in offspring at follow-up across the increasing number of parents with overweight and obesity at baseline (*P* for trend < 0.001).

Table 2 shows the parental weight change and risk of overweight and obesity in offspring. Compared with the paternal persistent normal group, the paternal persistent overweight and obesity group (odds ratio [95% confidence interval], OR [95% CI]: 3.22 [2.13, 4.87]) and the paternal incident overweight and obesity group (OR [95% CI]: 2.46 [1.58, 3.84]), but not the paternal reversion group, were more likely to report overweight and obesity in offspring at follow-up. Similar results were obtained for the association of maternal weight change with the incidence of overweight and obesity in offspring. Table S3 reports the results of sensitivity analyses stratified by the sex of offspring. In male offspring, the influence of paternal and maternal weight change on the incidence of overweight and obesity were similar to those reported above. In female offspring, compared with the paternal persistent normal group, the paternal persistent overweight and obesity group was more likely to report overweight and obesity in offspring at follow-up; however, we did not identify similar trends in the mother–female offspring weight association. Table S4 presents the results of the sensitivity analyses using the China overweight and obesity definition. In general, the results did not change substantially relative to the findings presented by Table 2.

**Table 2:** Parental weight change and risk of overweight and obesity in offspring

Weight status	Incidence of overweight and obesity in offspring, %	OR (95%CI)^a^	*P*
Paternal weight change			
Persistent normal weight (*n* = 2252)	3.8	Ref	
Reversion (*n* = 94)	7.5	1.74 (0.75–4.02)	0.198
Incident overweight and obesity (*n* = 306)	10.5	2.46 (1.58–3.84)	< 0.001
Persistent overweight and obesity (*n* = 311)	15.1	3.22 (2.13–4.87)	< 0.001
Maternal weight change			
Persistent normal weight (*n* = 2232)	4.9	Ref	
Reversion (*n* = 73)	2.7	0.58 (0.14–2.45)	0.457
Incident overweight and obesity (n = 306)	8.2	1.77 (1.10–2.82)	0.018
Persistent overweight and obesity (*n* = 352)	9.9	2.03 (1.34–3.09)	< 0.001

^a^Adjusted for sex, the length of follow-up, Han Nationality, and baseline characteristics (year of study entry, age, household asset score, urban residence and energy intake)

*OR* indicates odds ratio, *CI* indicates confidence interval

Ref indicated that the corresponding group was considered as “reference” group

Table 3 shows the combined influence of paternal and maternal weight change on the incidence of overweight and obesity in offspring at follow-up. Compared with children whose both parents remained normal weight, those whose one parent remained normal weight and the other developed overweight and obesity or whose one parent remained normal weight and the other remained to have overweight and obesity had higher risks of reporting overweight and obesity at follow-up (all *P*_s_ < 0.05). However, children whose both parents changed from overweight and obesity to normal weight or whose one parent changed from overweight and obesity to normal weight and the other remained normal weight did not report such higher risks (OR [95% CI]: 1.33 [0.51, 3.49]).

**Table 3:** Combinations of paternal and maternal weight change and risk of overweight and obesity in offspring

Weight change^b^	Incidence of overweight and obesity in offspring, %	OR (95%CI)^a^	*P*
Both parents remained normal weight (*n* = 1777)	3.4	Ref	
Both parents’ weights changed from overweight and obesity to normal weight/one parent’s weight changed from overweight and obesity to normal weight and the other remained normal weight (*n* = 110)	4.6	1.33 (0.51–3.49)	0.560
One parent remained normal weight and the other developed overweight and obesity (*n* = 412)	6.1	1.74 (1.06–2.86)	0.028
One parent remained normal weight and the other remained to have overweight and obesity (*n* = 413)	10.7	2.80 (1.83–4.28)	< 0.001
One parent’s weight changed from overweight and obesity to normal weight and the other remained to have overweight and obesity/one parent’s weight changed from overweight and obesity to normal weight and the other developed overweight and obesity (*n* = 52)	7.7	1.77 (0.59–5.29)	0.309
Both parents developed overweight and obesity/ Both parents remained to have overweight and obesity/One parent remained to have overweight and obesity and the other developed overweight and obesity (*n* = 199)	16.6	4.47 (2.76–7.23)	< 0.001

^a^ Adjusted for sex, the length of follow-up, Han Nationality, and baseline characteristics (year of study entry, age, household asset score, urban residence and energy intake)

^b^ Participants were categorized into six groups based on the combinations of paternal and maternal weight change (Table S1)

*OR* indicates odds ratio, *CI* indicates confidence interval

Ref indicated that the corresponding group was considered as “reference” group

## Discussion

In this study, parental overweight and obesity at baseline were significantly correlated with these conditions in offspring at follow-up. Compared with the persistent normal group, the persistent overweight and obesity group and the incident overweight and obesity group, but not the reversion group, were more likely to report overweight and obesity in offspring at follow-up, regardless of the father’s or mother’s condition. Compared with children whose both parents remained normal weight, those whose both parents changed from overweight and obesity to normal weight or whose one parent changed from overweight and obesity to normal weight while the other remained normal weight had no higher risks of reporting overweight and obesity at follow-up.

The findings of the present study are consistent with those of a previous study that reported that overweight and obesity during the parent’s course of life are associated with the risk of the same conditions in offspring aged 4–18 years [19]. A cohort study further pointed out that parental obesity during their offspring’s childhood is a primary determinant of obesity in adult offspring [20]. The 1958 British Birth Cohort and the Framingham Heart Study both reported the transmission of overweight and obesity across two generations and demonstrated that the significant association between parental and children’s overweight and obesity had strengthened over time [21, 22]. Our findings were also consistent with those of another study showing that children whose one or both parents had overweight and obesity were more likely to develop overweight and obesity than those whose both parents had normal weight [20, 22, 23].

The advantage of this study is that it clarifies the relationship between parental weight change and the incidence of overweight and obesity in offspring during the offspring’s childhood. Our findings are in accordance with a previous study showing that an excessive increase in the BMI of parents over 16 years of follow-up predicted the incidence of overweight and obesity in offspring [10]. The findings of the Framingham Heart Study also partly support our finding that parental obesity predicted weight gain and incident obesity in offspring [22]. The results of previous studies regarding mother–son, father–son, and father–daughter weight relationships are also similar to our findings [24–26]. Notably, studies have revealed significant correlations between the weight statuses of mothers and daughters [24–26]. However, no association between mothers’ weight change and the incidence of overweight and obesity in female offspring was observed in our study. This discrepancy is likely due to the differences in the sample size, age range, and ethnic composition of the study populations and the time points for measuring the parental and offspring weight and height. The Longitudinal Study of Australian Children indicates that fathers rather than mothers play an important role in the development of overweight and obesity in offspring, which is partly lined with our findings [27]. Further studies are needed to compare the relative associations of paternal and maternal weight with weight in offspring during different time periods [13].

Both our study and the Northern Finland Birth Cohort 1986 reported the combined associations between parental weight change and the incidence of overweight and obesity in offspring [10]. In contrast to a previous study, this study provided value information: compared with children whose both parents remained normal weight, those whose both parents changed from overweight and obesity to normal weight or whose one parent changed from overweight and obesity to normal weight while the other remained normal weight had no higher risks of reporting overweight and obesity at follow-up [10].

Some potential mechanisms may explain our findings. First, overweight and obesity are partly influenced by genetic determinants [19, 28]. A study indicated that assortative mating for obesity contributes to the risk of obesity in offspring and consequently to the obesity epidemic [29]. A UK sample of 5,092 twin pairs aged 8–11 years confirmed the high impact of genetic determinants on BMI [30]. Second, family members may share similar social environment, lifestyle, and dietary patterns [19, 22, 26, 31], given that parents shape the behavior patterns of their offspring and create a family environment. For example, parental eating preferences have been shown to have a crucial impact on the food choice and the consequent obesity risk of adolescents [26, 32]. Taken together, the literature suggests that parental obesity represents a surrogate marker of the influence of genetic determinants and shared environment, lifestyle, and dietary patterns on the obesity risk of their offspring [31].

The strengths of this study include the household-based design and longitudinal cohort that allowed us to directly measure parental weight over the long term and obtain high-quality data. Additionally, we also acknowledge several limitations in this study. First, the small sample size may have affected the results of our analyses. Further studies using larger cohorts are needed to verify our findings. However, our main results (e.g. association between parental overweight and obesity and these conditions in offspring) were consistent with a previous meta-analysis [13]. Second, the biological mechanism underlying the parent–offspring weight relationship was not determined in this study. However, our study and previous publications suggested that the biological mechanism associated with genetic determinants and shared environment, lifestyle, and dietary patterns should be determined [19, 22, 26, 28–31]. Third, our study was conducted in a Chinese cohort. The findings needed to be interpreted with caution in other population. However, a previous meta-analysis including participants from many countries confirmed our main results [13]. Finally, given the observational nature of this study, causal associations could not be established.

## Conclusions

In summary, this study showed that parental weight change was associated with the incidence of overweight and obesity in offspring. This finding emphasizes the importance of parental weight management in the prevention of overweight and obesity in offspring.

## Supplementary Information

**Additional file 1: Table S1** Groups based on the combinations of paternal and maternal weight change. **Table S2** Parental baseline weight and risk of overweight and obesity in offspring at follow up. **Table S3** Parental weight change and risk of overweight and obesity in male and female offspring. **Table S4** Parental weight change and risk of overweight and obesity in offspring using China overweight and obesity definition.

## Data Availability

The datasets used and/or analyzed during the current study are available from the corresponding author on reasonable request.

## Abbreviations

BMI: Body mass index
CHNS: China Health and Nutrition Survey
