# Supplementary material for: Influence of parental weight change on the incidence of overweight and obesity in offspring
Source: BMC Pediatr. 2022 Jun 7;22:330. doi: 10.1186/s12887-022-03399-8 (PMC9172004; doi:10.1186/s12887-022-03399-8)
Supplement: Supplementary file 1 — Additional file 1: Table S1 Groups based on the combinations of paternal and maternal weight change. Table S2 Parental baseline weight and risk of overweight and obesity in offspring at follow up. Table S3 Parental weight change and risk of overweight and obesity in male and female offspring. Table S4 Parental weight change and risk of overweight and obesity in offspring using China overweight and obesity definition. [file 12887_2022_3399_MOESM1_ESM.docx]

| Table S1. Groups based on the combinations of paternal and maternal weight change | | |
| --- | --- | --- |
| Paternal weight change | Maternal weight change | Combinations of parental weight change |
| Paternal persistent normal weight | Maternal persistent normal weight | Both parents remained normal weight |
| Paternal reversion | Maternal persistent normal weight | Both parents’ weights changed from overweight and obesity to normal weight/one parent’s weight changed from overweight and obesity to normal weight and the other remained normal weight |
| Paternal incident overweight and obesity | Maternal persistent normal weight | One parent remained normal weight and the other developed overweight and obesity |
| Paternal persistent overweight and obesity | Maternal persistent normal weight | One parent remained normal weight and the other remained to have overweight and obesity |
| Paternal persistent normal weight | Maternal reversion | Both parents’ weights changed from overweight and obesity to normal weight/one parent’s weight changed from overweight and obesity to normal weight and the other remained normal weight |
| Paternal reversion | Maternal reversion | Both parents’ weights changed from overweight and obesity to normal weight/one parent’s weight changed from overweight and obesity to normal weight and the other remained normal weight |
| Paternal incident overweight and obesity | Maternal reversion | One parent’s weight changed from overweight and obesity to normal weight and the other remained to have overweight and obesity/one parent’s weight changed from overweight and obesity to normal weight and the other developed overweight and obesity |
| Paternal persistent overweight and obesity | Maternal reversion | One parent’s weight changed from overweight and obesity to normal weight and the other remained to have overweight and obesity/one parent’s weight changed from overweight and obesity to normal weight and the other developed overweight and obesity |
| Paternal persistent normal weight | Maternal incident overweight and obesity | One parent remained normal weight and the other developed overweight and obesity |
| Paternal reversion | Maternal incident overweight and obesity | One parent’s weight changed from overweight and obesity to normal weight and the other remained to have overweight and obesity/one parent’s weight changed from overweight and obesity to normal weight and the other developed overweight and obesity |
| Paternal incident overweight and obesity | Maternal incident overweight and obesity | Both parents developed overweight and obesity/both parents remained to have overweight and obesity/one parent remained to have overweight and obesity and the other developed overweight and obesity |
| Paternal persistent overweight and obesity | Maternal incident overweight and obesity | Both parents developed overweight and obesity/both parents remained to have overweight and obesity/one parent remained to have overweight and obesity and the other developed overweight and obesity |
| Paternal persistent normal weight | Maternal persistent overweight and obesity | One parent remained normal weight and the other remained to have overweight and obesity |
| Paternal reversion | Maternal persistent overweight and obesity | One parent’s weight changed from overweight and obesity to normal weight and the other remained to have overweight and obesity/one parent’s weight changed from overweight and obesity to normal weight and the other developed overweight and obesity |
| Paternal incident overweight and obesity | Maternal persistent overweight and obesity | Both parents developed overweight and obesity/both parents remained to have overweight and obesity/one parent remained to have overweight and obesity and the other developed overweight and obesity |
| Paternal persistent overweight and obesity | Maternal persistent overweight and obesity | Both parents developed overweight and obesity/both parents remained to have overweight and obesity/one parent remained to have overweight and obesity and the other developed overweight and obesity |

| Table S2. Parental baseline weight and risk of overweight and obesity in offspring at follow up | | | |
| --- | --- | --- | --- |
| Weight status | Incidence of overweight and obesity in offspring, % | OR (95%CI) † | *P* |
| Paternal baseline weight |  |  |  |
| Normal weight (n=2558) | 4.6 | ref |  |
| Overweight and obesity (n=405) | 13.3 | 2.37 (1.63, 3.45) | <0.001 |
| Maternal baseline weight |  |  |  |
| Normal weight (n=2538) | 5.3 | ref |  |
| Overweight and obesity (n=425) | 8.7 | 1.65 (1.11, 2.45) | 0.014 |
| Number of parental baseline overweight and obesity |  |  |  |
| 0 (n=2243) | 4.2 | ref |  |
| 1 (n=610) | 10.7 | 2.29 (1.61, 3.26) | <0.001 |
| 2 (n=110) | 11.8 | 2.53 (1.32, 4.88) | 0.005 |
| Trend* |  | 1.85 (1.43, 2.39) | <0.001 |

†Adjusted for sex, the length of follow-up, Han Nationality, and baseline characteristics (year of study entry, age, household asset score, urban residence and energy intake).

*We considered the number of parental baseline overweight and obesity as a continuous variable and used the covariate-adjusted logistic model to evaluate the trend in the incidence of overweight and obesity in offspring.

OR indicates odds ratio; CI indicates confidence interval.

| Table S3. Parental weight change and risk of overweight and obesity in male and female offspring | | | | | | | |
| --- | --- | --- | --- | --- | --- | --- | --- |
|  | Overweight and obesity in male offspring | | |  | Overweight and obesity in female offspring | | |
| Weight status | n/N | OR (95% CI)† | *P* |  | n/N | OR (95% CI)† | *P* |
| Paternal weight change |  |  |  |  |  |  |  |
| Persistent normal weight | 50/1156 | Ref |  |  | 35/1096 | Ref |  |
| Reversion | 4/46 | 1.71 (0.56, 5.18) | 0.344 |  | 3/48 | 2.09 (0.57, 7.60) | 0.264 |
| Incident overweight and obesity | 21/153 | 2.80 (1.59, 4.94) | <0.001 |  | 11/153 | 1.87 (0.88, 3.97) | 0.103 |
| Persistent overweight and obesity | 33/181 | 3.62 (2.16, 6.08) | <0.001 |  | 14/130 | 2.80 (1.37, 5.73) | 0.005 |
| Maternal weight change |  |  |  |  |  |  |  |
| Persistent normal weight | 66/1169 | Ref |  |  | 43/1063 | Ref |  |
| Reversion | 1/30 | 0.52 (0.07, 4.13) | 0.540 |  | 1/43 | 0.82 (0.11, 6.25) | 0.847 |
| Incident overweight and obesity | 17/159 | 2.00 (1.11, 3.59) | 0.021 |  | 8/147 | 1.60 (0.71, 3.61) | 0.259 |
| Persistent overweight and obesity | 24/178 | 2.40 (1.42, 4.05) | 0.001 |  | 11/174 | 1.75 (0.85, 3.59) | 0.130 |

†Adjusted for Han Nationality, the length of follow-up, and baseline characteristics (year of study entry, age, urban residence, energy intake, and household asset score).

OR indicates odds ratio; CI indicates confidence interval.

| Table S4. Parental weight change and risk of overweight and obesity in offspring using China overweight and obesity definition | | | |  |
| --- | --- | --- | --- | --- |
| Weight status | Incidence of overweight and obesity in offspring, % | OR (95% CI)† | *P* |  |
| Paternal weight change |  |  |  |  |
| Persistent normal weight (n=1325) | 3.0 | Ref |  |  |
| Reversion (n=69) | 8.7 | 2.48 (0.97, 6.35) | 0.058 |  |
| Incident overweight and obesity (n=217) | 7.4 | 2.23 (1.19, 4.17) | 0.012 |  |
| Persistent overweight and obesity (n=294) | 9.2 | 2.77 (1.61, 4.76) | <0.001 |  |
| Maternal weight change |  |  |  |  |
| Persistent normal weight (n=1248) | 3.4 | Ref |  |  |
| Reversion (n=80) | 3.8 | 1.17 (0.34, 3.96) | 0.806 |  |
| Incident overweight and obesity (n=222) | 6.3 | 1.88 (0.98, 3.58) | 0.056 |  |
| Persistent overweight and obesity (n=355) | 8.5 | 2.51 (1.52, 4.14) | <0.001 |  |
| †Adjusted for sex, the length of follow-up, Han Nationality, and baseline characteristics (year of study entry, age, household asset score, urban residence and energy intake).  OR indicates odds ratio; CI indicates confidence interval. | | | |  |
|  |  |  |  |  |
|  |  |  |  |  |
